# Spatio-temporal evolution and driving factors of green innovation efficiency in the Chinese urban tourism industry based on spatial Markov chain

**DOI:** 10.1038/s41598-024-61226-9

**Published:** 2024-05-09

**Authors:** Zhenjie Liao, Shan Liang, Xuanfei Wang

**Affiliations:** 1 School of Management, Guangzhou Huashang College, Guangzhou, 511300 China; 2 School of Economics, Guangzhou City University of Technology, Guangzhou, 510800 China; 3 School of Information, Guangdong University of Finance and Economics, Guangzhou, China

**Keywords:** Tourism, Green innovation efficiency, Spatio-temporal evolution, Driving factors, Spatial Markov chain, Ecology, Environmental social sciences

## Abstract

Green innovation in the tourism industry is a sustainable development concept for resource conservation and environmental optimization. The effective measurement of green innovation efficiency in the tourism industry and an accurate understanding of its spatial relationship was significantly important for promoting its sustainable development. Using the SBM-undesirable model, kernel density estimation, and a spatial Markov chain, we explored the spatio-temporal evolution characteristics and influencing mechanisms of urban tourism green innovation efficiency (TGIE) in China between 2000 and 2020. We found that (1) the temporal and spatial changes of TGIE were generally at a lower than medium level and fluctuated throughout country, with a transition in the east, collapse in the middle, and stagnation in the northeast. (2) The dynamic evolution of TGIE always exhibited polarization, but regional coordination was gradually enhanced with strong stability, although it was difficult to achieve leap-forward development. The cities with spatial upward transfer were concentrated mainly in the central and western region and while there were few cities with a downward adjustment, there were obvious asymmetrical spatial spillover effects. (3) The driving factors of TGIE were the overall economic level, industrial structure, government regulation, and education level. These factors had a significant positive relationship with TGIE, while the degree of opening up to the outside world has no significant effect, but the degree of influence, mechanism, and conditions of each factor were strongly regional.

## Introduction

The pressing issues of resource consumption and environmental pollution have shoved the world into a critical juncture, necessitating immediate reflection and action^1^. Amidst the growing challenges of population growth, resource scarcity, and environmental constraints, China’s economic development has unfolded a pattern of extensive growth marked by excessive input, consumption, emissions, pollution, and recycling challenges. This calls for a paradigm shift towards green development and technological innovation to drive industrial transformation, production mode shifts, and efficiency enhancements^2^. The synergy of environmental concerns and resource limitations has propelled sustainable resource utilization and pollution reduction to the forefront of global agendas. Green innovation, with its core objectives of conservation, pollution reduction, and sustainable economic growth, has emerged as a pivotal strategy for countries striving to revamp industrial structures, enhance competitiveness, and improve environmental performance^3–5^. In the tourism sector, green innovation holds significant potential in curbing resource use and pollution, steering the industry towards high-quality development through sustainable resource management and eco-friendly practices^6^. Regionally coordinated development is also a critical consideration. Economic interlinkages and interactions are increasingly influential in governance and decision-making^7^. Regional cooperation in market sourcing, resource development, and tourism has become more interdependent^8^. The “network paradigm” has shifted urban research focus to the multi-scale relationships and functions of networks^9^, with cities now organized in a network dominated by temporal proximity^10^. This challenges the traditional linear model of regional economic spaces and raises questions about the spatial evolution patterns of green innovation in urban tourism and the impact of network structure changes on regional tourism green innovation development^11^.

The concept of green innovation, originally proposed by Fussle^12^ as new products and processes that add value while reducing environmental impact, has been expanded by scholars to encompass marketing methods, production processes, organizational structures, and institutional configurations^13^. Research now adopts an interdisciplinary approach, integrating economics, geography, management, environmental science, and sociology^14^. Also known as eco-innovation, environmental innovation, and sustainable innovation^15^, green innovation underscores the dual significance of innovation and environmental benefits^16^. Green innovation efficiency measures the effective use of input factors in production, factoring in environmental pollution and resource consumption^17^. Studies have examined the status, influencing factors, and spillover effects of green innovation efficiency at regional, industrial, and enterprise levels^18–20^. In the tourism industry, green innovation involves resource conservation and eco-friendliness through sustainable development-oriented innovative activities, including product, process, management, and institutional innovation^21^. International research highlights the impact of green innovation on various tourism sectors, labor productivity, and business performance^22,23^, as well as the role of organizational culture, scale, and environmental attitudes in fostering green innovation practices that enhance organizational performance and competitive advantage^24,25^. Chinese scholars have focused on the green innovation program selection, theory building, and efficiency measurement to improve the sustainability and quality of the tourism industry^26,27^, revealing higher green innovation efficiency in the eastern region compared to central and western China^28^. With national emphasis on green development and innovation-driven concepts, research on tourism industry eco-efficiency has evolved to consider economic, ecological, and innovative benefits through input–output relationships, innovation activities, and factor inputs^29,30^. Current research predominantly employs data envelopment analysis and other methods to evaluate efficiency and explore regional differences and spatial spillover effects^31^.

Despite substantial academic progress in green tourism innovation, there is room for further advancement. Research has largely concentrated on national and provincial scales, overlooking the city scale as a crucial starting point for investigating spatial heterogeneity in tourism green innovation efficiency. In this study, we build upon previous research results^32,33^ and take Chinese prefecture-level cities as a research object to construct a theoretical framework of green innovation in tourism and establish an evaluation system of green innovation efficiency in tourism that combines the SBM-undesirable model to measure the green innovation efficiency of China's tourism industry from 2000 to 2020. Our study applied the KDE kernel density estimation^34,35^, the coefficient of variation^36^, and a spatial Markov chain model to reveal its spatio-temporal evolution characteristics, as well as used a panel data regression model to explore the driving factors of green innovation efficiency to provide a scientific reference for the theory and practice of green innovation in tourism. This study attempted to answer the following questions: (1) What is the temporal change in the overall green innovation efficiency of urban tourism in China? (2) What are the spatial distribution characteristics of the green innovation efficiency in Chinese cities? (3) What are the mechanisms of each driving factor influencing the green innovation efficiency of urban tourism in China? (4) Is there spatial spillover effect? By answering these questions, this study proposes a relevant decision-making basis for improving the green innovation efficiency of the Chinese urban tourism industry. The innovations of this study include: (1) The SBM-DEA model with non-expected parameters was used to measure the green innovation efficiency of China's urban tourism industry, which improves on existing studies that have not fully considered the impact of non-expected output on the green innovation efficiency and accurately measures the current level of green innovation in China's urban tourism industry. (2) Analysis of the green innovation development track of China's urban tourism industry from multiple spatio-temporal perspectives that provide empirical support for further understanding of the green innovation development track of China’s urban tourism industry. (3) We use analysis of the economic development level, industrial structure, technological innovation level, government regulation, opening to the outside world and education level to explore the factors that affect the green innovation efficiency of China's urban tourism industry, and provide path support for further promotion of the green innovation development of China's urban tourism industry.

## Research methodology and data sources

### Research methodology

#### Evaluation model of green innovation efficiency in tourism

The green innovation efficiency (TGIE) refers to the green benefits achieved through innovation activities and innovation factor input; that is, the proportional relationship among the innovation factor input, resources, and environmental output. The higher the green innovation efficiency, the more matched the innovation activities and innovation factors are with the resources and environmental benefits they accrue^32,37,38^. The TGIE reflects the degree of the contribution per unit innovation input to innovation output, with the objective measurement reducing input redundancy and optimize the allocation of innovation resources^39^. The basic idea of traditional DEA model evaluation is to produce as much output as possible with the smallest amount of input; however, it does not consider the “undesirable output”, such as environmental pollution and resource consumption^40^. We evaluated the green innovation efficiency of China's tourism industry by incorporating a super-efficiency slack-based measure (SBM) model with non-expected output to solve the deviation problem of efficiency measurements caused by the traditional DEA model without considering relaxation variables^41^. Considering that resource endowment is the basic condition for tourism industry development, it is more objective and reasonable to include it in the efficiency evaluation system. Based on previous research^33^, we included human, capital, and resources as input indicators, and the tourism economy and the non-expected output of tourism environmental pollution as the expected output indicators. An evaluation index system for the green innovation efficiency of China’s urban tourism industry was constructed to measure the allocation level of tourism input–output under the conditions of resource consumption and environmental pollution (Table 1). The scientific selection of input and output indicators was the basis for measuring the efficiency of green innovation in the tourism industry. The specific measurement indicators were selected as follows:

1. Input indices: Existing studies mainly considered the input index of green innovation efficiency in terms of the number of scientific researchers and the investment in scientific research funds; however, these studies are more suitable for scientific research and independent innovation. The tourism industry is labor intensive and has a relatively high dependence on the quality of employees and requires the input of material resources. This study selected corresponding indicators to measure the input of green innovation from the perspectives of human, material, and financial resources in the tourism industry. First, human capital is the leading force in innovation activities. As the main body and executors of innovation activities, tourism researchers can represent the innovation level of the regional tourism industry to a certain extent. Therefore, several tourism researchers have been selected to represent the human input index of green innovation efficiency. Second, capital investments are the basic condition for conducting green innovation activities and can determine the success of green innovation as well as represent the institutional environment for tourism green innovation. We selected tourism research funds to represent capital investments.
2. Output indices: Green innovation efficiency differs from traditional technological innovation efficiency with economic benefits. This is the green process of innovation efficiency and the quality assessment of innovation development after considering energy consumption and environmental pollution. Therefore, the output variables in this study included expected and non-expected outputs. The expected output was used to measure the economic benefits of tourism obtained in the process of green innovation, and total tourism income was selected to represent this output. Tourism wastewater discharge, sulfur dioxide discharge, and soot discharge were selected to reflect tourism environmental pollution and used as unexpected output indicators.

**Table 1:** The TGIE evaluation system.

Primary indicator	Secondary indicators	Tertiary indicators	Unit
Throw oneself into	Manpower inputs	Number of tourism researchers	Ten thousand people
	Capital investment	Expenditure on tourism research	Ten thousand Yuan
	Resource inputs	Total water supply for tourism	Ten thousand tons
		Total electricity consumption in tourism	Ten thousand kilowatt-hours
		Total gas supply for tourism	Ton
Outputs	Expected outputs	Gross tourism receipts	Hundred million yuan
	Non-expected outputs	Wastewater discharge from tourism	Ten thousand tons
		Sulphur dioxide emissions from tourism	Ton
		Smoke and dust emissions from tourism	Ton

#### SBM-undesirable modeling

The DEA model has a certain error in measuring the efficiency of non-desired outputs and does not consider ineffective DMU slack variables. While the tourism industry is developing economic, social, and other desired outputs, it also produces carbon emissions, wastewater, exhaust gas, and other non-desired outputs. To more accurately measure the green innovation efficiency of the tourism industry in the presence of non-desired outputs, we applied the slack-based model (SBM)-undesirable model, which can effectively repair the problems of ineffective DMU slack variable portions and non-desired outputs.

1 $$\rho = {\text{min}}\frac{{1 - \frac{1}{N}\sum\nolimits_{n = 1}^{N} {\left(S_{n}^{x} /x_{p^{\prime}n}^{t^{\prime}} \right)} }}{{1 + \frac{1}{M + 1}\left( {\sum\nolimits_{m = 1}^{M} {S_{m}^{y} } } \right./y_{p^{\prime}m}^{t^{\prime}} + \left. {\sum\nolimits_{i = 1}^{I} {S_{i}^{b} /b_{p^{\prime}i}^{t^{\prime}} } } \right)}}$$

2 $$s.t.\sum\limits_{t = 1}^{T} {\sum\limits_{p = 1}^{P} {z_{p}^{t} } } x_{pn}^{t} + S_{n}^{x} = x_{p^{\prime}n}^{t^{\prime}} ,\; (n = 1,2 \ldots ,N),\; \sum\limits_{t = 1}^{T} {\sum\limits_{p = 1}^{P} {z_{p}^{t} } } y_{pn}^{t} - S_{m}^{y} = y_{p^{\prime}m}^{t^{\prime}} ,\; (m = 1,2 \ldots ,M)$$

3 $$\sum\limits_{t = 1}^{T} {\sum\limits_{p = 1}^{P} {z_{p}^{t} } } x_{pi}^{t} + S_{i}^{b} = b_{p^{\prime}i}^{t^{\prime}} ,\; (i = 1,2 \ldots ,I),\; z_{p}^{t} \ge 0,s_{n}^{x} \ge 0,s_{m}^{y} \ge 0,s_{i}^{b} \ge 0,\; (p = 1,2, \ldots ,P)$$

where* ρ* is the target efficiency value; *N*, *M*, *I* are the number of inputs, desired outputs, and non-desired outputs, respectively; n = [1, *N*], m = [1, *M*], and i = [1, *I*]; *p* is the decision unit; *t* is the time; and are the slacks of inputs, desired outputs, and non-desired outputs, respectively. Additionally, are the input–output values of the decision unit *p*′ based on the inputs, desired outputs, and non-desired outputs in the time period *t*′ is the weight vector of the decision unit, and are the values of the inputs and outputs based on the inputs, desired outputs, and non-desired outputs at time* t* of the decision cell, respectively; and is the weight vector of the decision cell.

#### Nonparametric kernel density estimation

Kernel density estimation (KDES) was used to estimate the smooth empirical probability density function^42^, which is a nonparametric test that is a common spatial analysis technique to characterize the spatial distribution intensity of geographic events. Let the density function of the random variable *f*(*x*) = *f*(*x*_1_, *x*_2_, *x*_3_), *x*_1_, *x*_2_, *x*_n_ reflect independently distributed samples, where the probability density of the random variable at point *x* is estimated by the formula:

4 $$f(x) = \frac{1}{Nh}\sum\limits_{i = 1}^{n} {K\left(\frac{{X_{i} - x}}{h}\right)}$$

where *N* is the total number of samples (297 cities in China), *K* is the stochastic kernel function, *h* is the density estimation bandwidth. The larger the bandwidth, the smoother the density estimation, and the larger the deviation.

#### Spatial Markov chains

The Spatial Markov chain was formed by combining spatial autocorrelation and the traditional Markov chain^43^. By comparing the transfer probability of the corresponding elements in the non-spatial matrix with the spatial matrix, as well as the relationship among the neighboring regions, it can better reveal the spatial interaction relationship with the neighboring regions when a certain attribute of a region changes, as well as possible spatial spillover effects. TGIE may be affected by the green innovation efficiency of the neighboring tourism industry and its spatio-temporal evolution requires spatial factors. The calculation principle was as follows: continuous data were discretized into *n* types, the transfer between all types in different periods constituted an *n* × *n* transfer probability matrix, and the probability of transferring *f*_*ij*_ from type *i* in period *t* to type *j* in the next period was *f*_*ij*_ = *g*_*ij*_/*g*_*i*_, where *g*_*ij*_ is the number of regions that were transferred from state *i* to state *j* from period *t* to the next period in the study period, *gi* is the probability that all regions in period *t* were transferred from state *i* to state *j* in the study period, and *gi* is the probability that all regions in period *t* were transferred from state *i* to state* j* in the study period. The total number (in number) of all period *t* regions in state *i* during the study period. If the initial year type was *i* and the next period type as still *i*, the region type was considered as “smooth”. If the value of the region attribute increased in the next period, the region type was considered as an “upward shift”, and vice versa, was a “downward shift”. The spatial Markov chain was conditioned on the spatial lag of region *i* in the initial year and the traditional *n* × *n* transfer probability matrix was reduced into *m n* × *n* conditional transfer probability matrices and for region *i*, the neighborhood was *j.* The spatial lag of the region is calculated as:

where *p*_*i*_ is the original attribute value of region *i*, *W*_*ij*_ is the spatial lag weight, and the proximity criterion was defined as adjacent regions *i* and *j* with their values = 1. Otherwise, this value is zero when *i* = *j* and *w*_*ij*_ = 0.

### Data sources

The data in this study were collected from the China Tourism Statistical Yearbook 2001–2021, China Culture and Tourism Statistical Yearbook 2021, National Data (https://data.stats.gov.cn), the National Database of the Bureau of Statistics of the People’s Republic of China, and the statistical yearbooks of cities on the Chinese mainland, China Energy Statistical Yearbook, and China Fixed Asset Investment Statistical Yearbook from to 2001–2021 for China cities, China Energy Statistical Yearbook, and China Fixed Asset Investment Statistical Yearbook. Given the principle of data availability, Hong Kong, Macao, and Taiwan were excluded.

## Analysis of results

### Characterizing the time evolution of green innovation efficiency in the Chinese tourism industry

**Figure 1:**
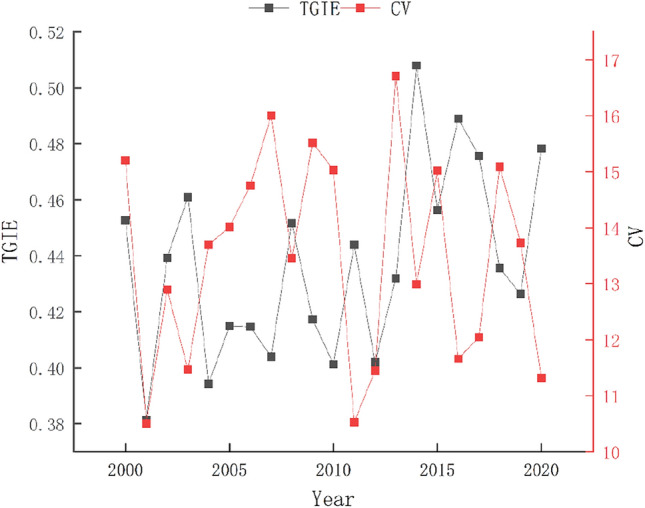
The temporal evolution of TGIE in China from 2000 to 2020.

According to the changes of green innovation efficiency and the regional coefficient of variation (CV) of tourism industry for 297 Chinese cities from 2000 to 2020 (Fig. 1), we found that the green innovation efficiency of China's tourism industry was generally at a medium level, showing the “W” type of “decreasing–rising–decreasing–rising”.

### Characterization of the spatial evolution of green innovation efficiency in the Chinese tourism industry

**Figure 2:**
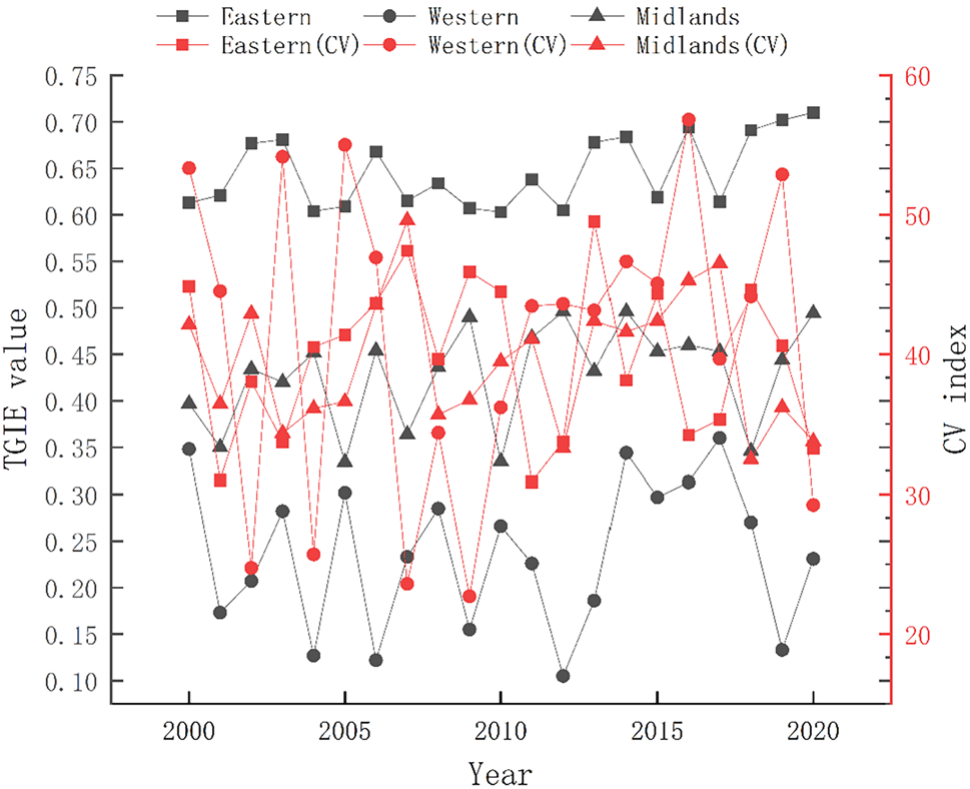
The differences in TGIE among the three study regions in China from 2000 to 2020.

At the regional level, the green innovation efficiency of China's tourism industry from 2000 to 2020 had a decreasing distribution pattern that followed “east–middle–west”; and obvious pattern of evolution and internal differences among the three major regions (Fig. 2). The temporal evolution patterns and internal differences among the three regions were also evident (Fig. 2). The degree imbalance was western region > eastern region > central region, but the degree of imbalance in each region converged such that the eastern region had a smaller change and the central and western region showed a “wavy” and inverted “V” pattern of reduction. The eastern region was always the leading region in tourism green innovation, with abundant high-quality tourism resources, high economic level, social welfare and technological innovation, and better tourism reception and service quality. However, it also had high-intensity urbanization and industrialization activities, and the tourism activities were not completely decoupled from carbon emissions and energy consumption. The central region was relatively balanced in development and had rich tourism resources; however, the scattered distribution of attractions, poor accessibility, low income of urban and rural residents, and insufficient demand for international tourism produced only medium level economic benefits from tourism. Additionally, the TGIE rapidly increased under the driving force of a sound tourism policy system, a strong demand for tourism, and the practice of ecological civilization construction. The western region had good resource and energy advantages but most of the cities were in remote and border areas of China that have low urbanization and economic levels, a fragile ecological environment, an irrational high density development pattern, high consumption and high emission of energy industry, weak technological innovation capacity and limited tourism hospitality, and the TGIE was lagging.

**Figure 3:**
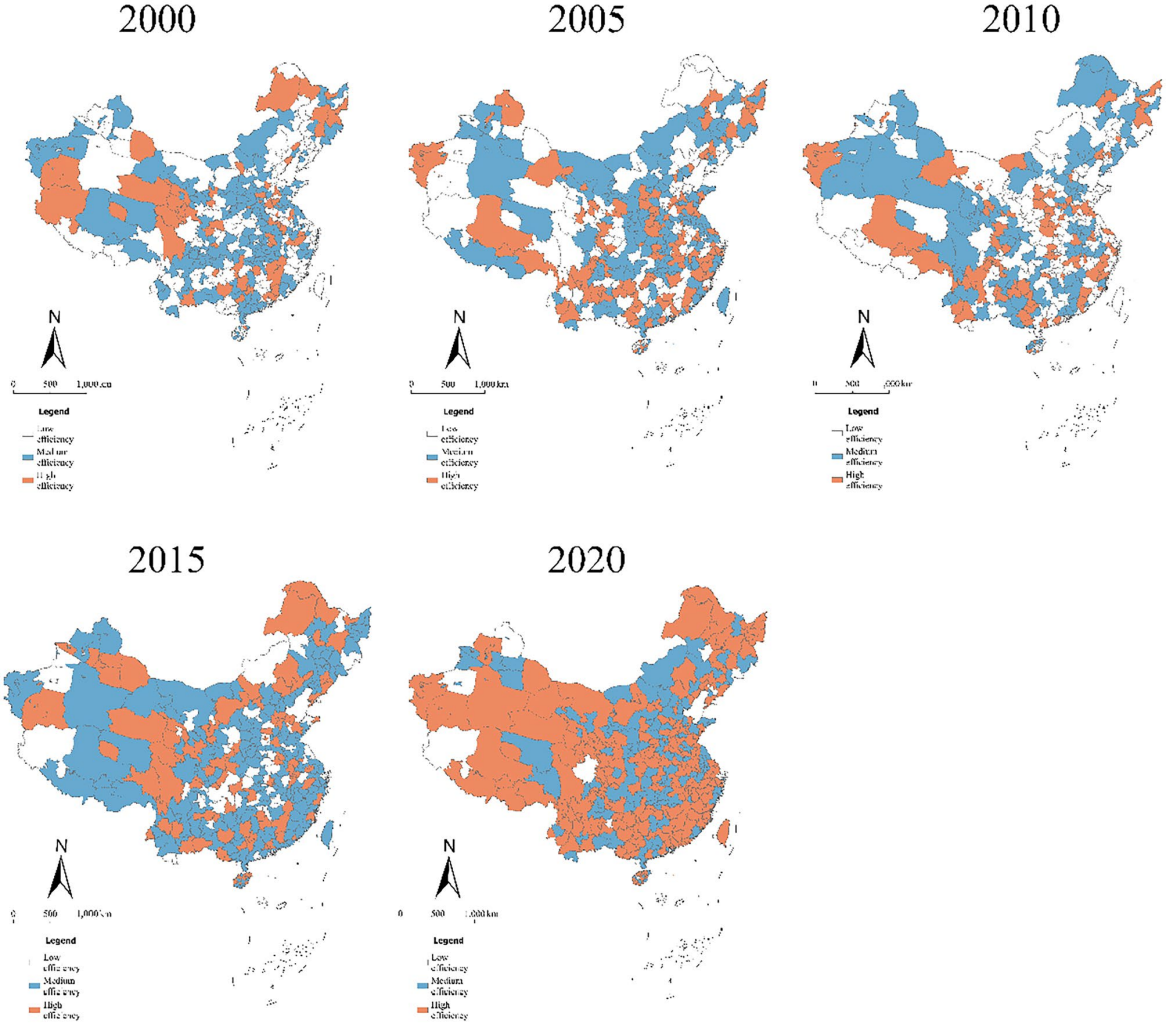
The spatial distribution of TGIE in China from 2000 to 2020.

In recent years, due to the influence of national policies, market demand, and the change in development concepts, the regional industrial structure and development mode have been optimized and the level of tourism development has substantially increased. Together with good natural ecosystems, the level of TGIE gradually improved. Using the division standard of efficiency measurement described previously^44^, the TGIE at the provincial level was divided into three levels of low, medium, and high efficiency with critical values of 0.33 and 0.66. The TGIE grading results in 2000, 2005, 2010, 2015, and 2020 were visualized and mapped using ArcGIS 10.1 software (Fig. 3). Overall, the green innovation efficiency of China's tourism industry developed in a clustered and continuous manner, with the low, medium, and high efficiency changing from a “pyramid” to a “diamond” structure with an obvious distribution of zones. The number of high-efficiency areas was relatively small and gradually increased over time, spreading from the eastern coast to the middle and upper reaches of the Yangtze River. Most high-efficiency regions had strong locations, resources, and policy advantages, and gradually realized intensive and efficient development of tourism through advanced technology and optimization of industrial structure. Greater progress was made in environmental governance and effective energy conservation and emission reduction in tourist hotels and attractions. Additionally, the proportion of resident trips and the share of tourism consumption expenditure gradually increased, with higher ecological, environmental, and socio-economic benefits in 2000. Medium efficiency was distributed in the east and southwest over time, especially in 2020, and were mainly clustered in the southeast. Low-efficiency cities were mainly distributed on both sides of the Hu Huanyong line and gradually decreased. Low-efficiency regions had a low level of tourism economic development, insufficient R&D, and innovation capacity of green tourism products, coupled with resources and location constraints. The regional industrial structure was heavy, resulting in excessive regional energy development, high carbon emissions, slow tourism industry development, and relatively low green development efficiency.

### Characterizing the dynamic evolution of green innovation efficiency in the tourism industry

#### Kernel density estimation

**Figure 4:**
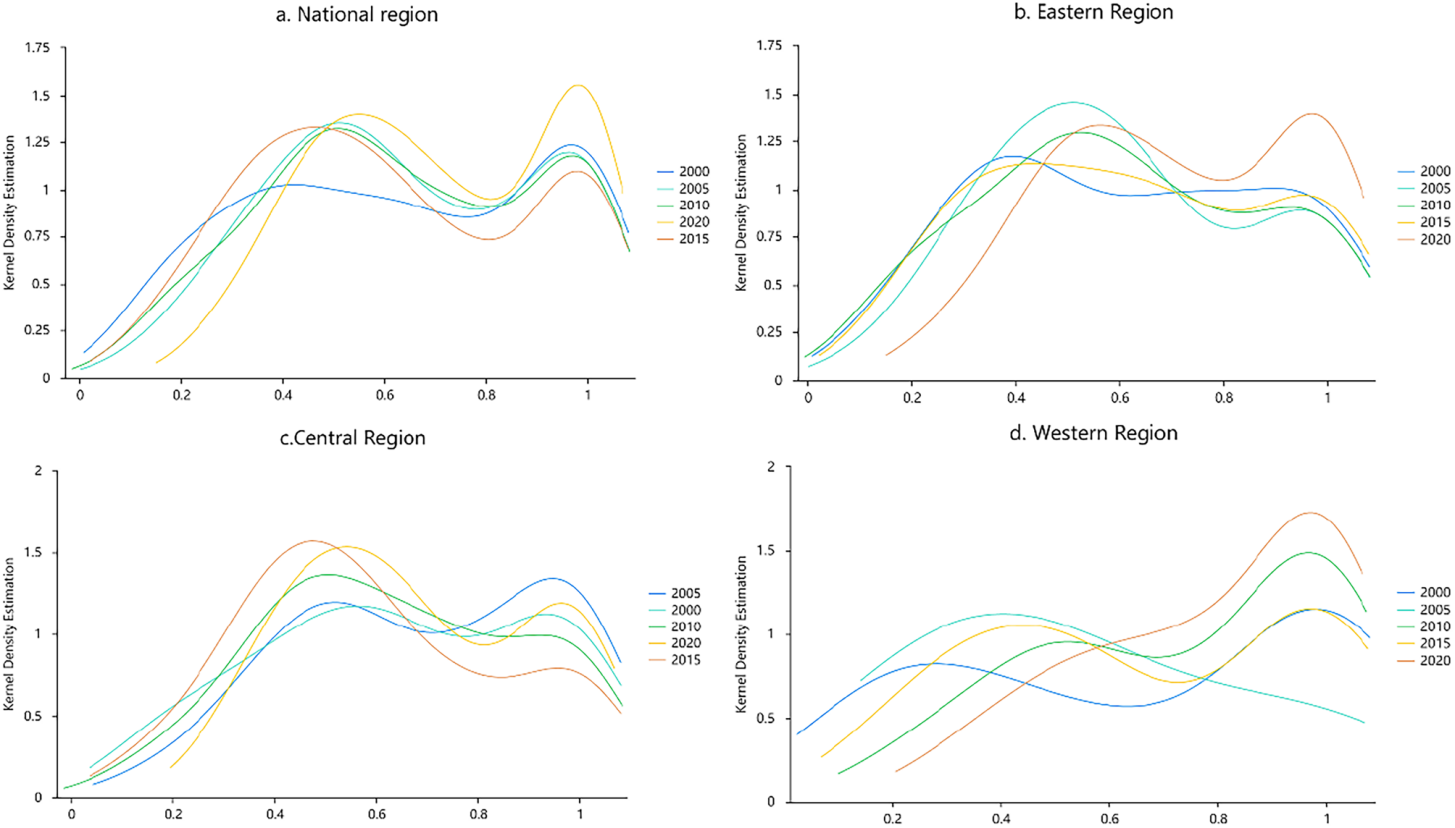
The kernel density estimation of TGIE in China from 2000 to 2020.

Combined with the time-series analysis, the kernel density estimation curves for the entire country and the three regions were obtained for 2000, 2005, 2010, 2015, and 2020 study periods (Fig. 4). Overall, the kernel density distribution curve shifted to the right, indicating that the TGIE had a more pronounced growth during the sample period and that the tourism economic-social-ecological system equilibrium gradually increased (Fig. 4a). The shape of the curve changed from a sharp peak to a broad peak as the height of the wave decreased; the width widened and the kurtosis gradually flattened, indicating that the cities with higher TGIE increased annually and the inter-provincial gap narrowed. The high levels of TGIE clustered in the eastern region and the advantages of good location, resources, population, and policies improved the level of green innovation in tourism. There were significant differences in the level of TGIE in the central and western region, which largely depended on the local resources and the guidance and support of policies.

#### Characteristics of spatio-temporal shifts

Although kernel density estimation can better portray the overall pattern of regional TGIE development and change, it is difficult to visualize the changes in relative positions and future development trends in the evolution of TGIE in different regions. The spatial Markov chain is divided into several small regions in space, and the state transfer in each small region satisfies the Markov property, that is, it is only related to the previous state. The state transfer between different small areas takes into account the influence of location, so it is more suitable for the actual situation. Therefore, a spatial Markov chain was used to explore the internal mobility and stability status of the national TGIE. First, we used the reference to the spatio-temporal distribution characteristics of provincial TGIE to identify three types of conditions, which were represented by k = 1, 2, and 3 based on the provincial TGIE class (0–0.33 for low efficiency, 0.34–0.66 for medium efficiency, and 0.67–1.00 for high efficiency). Second, to understand the stage-by-stage changes in TGIE, the whole study period was divided into 2000–2005, 2005–2010, 2010–2015, and 2015–2020 sub-periods. Finally, using the principle of traditional Markov chain, the 2000–2020 TGIE traditional Markov chain transfer matrix was constructed (Table 2).

**Table 2:** The Markov transfer matrix for TGIE in China from 2000 to 2020.

Typology	1	2	3	Typology	1	2	3
2000–2005				2005–2010			
1	0.030	0.072	0.063	1	0.375	0.092	0
2	0.666	0.062	0.074	2	0	0.338	0.095
3	0.073	0.099	0	3	0.089	0	0.333
2010–2015				2015–2020			
1	0.040	0.096	0.064	1	0.225	0.088	0.074
2	0.702	0.065	0.015	2	0.086	0.163	0.426
3	0.683	0	0	3	0	0	0

**Figure 5:**
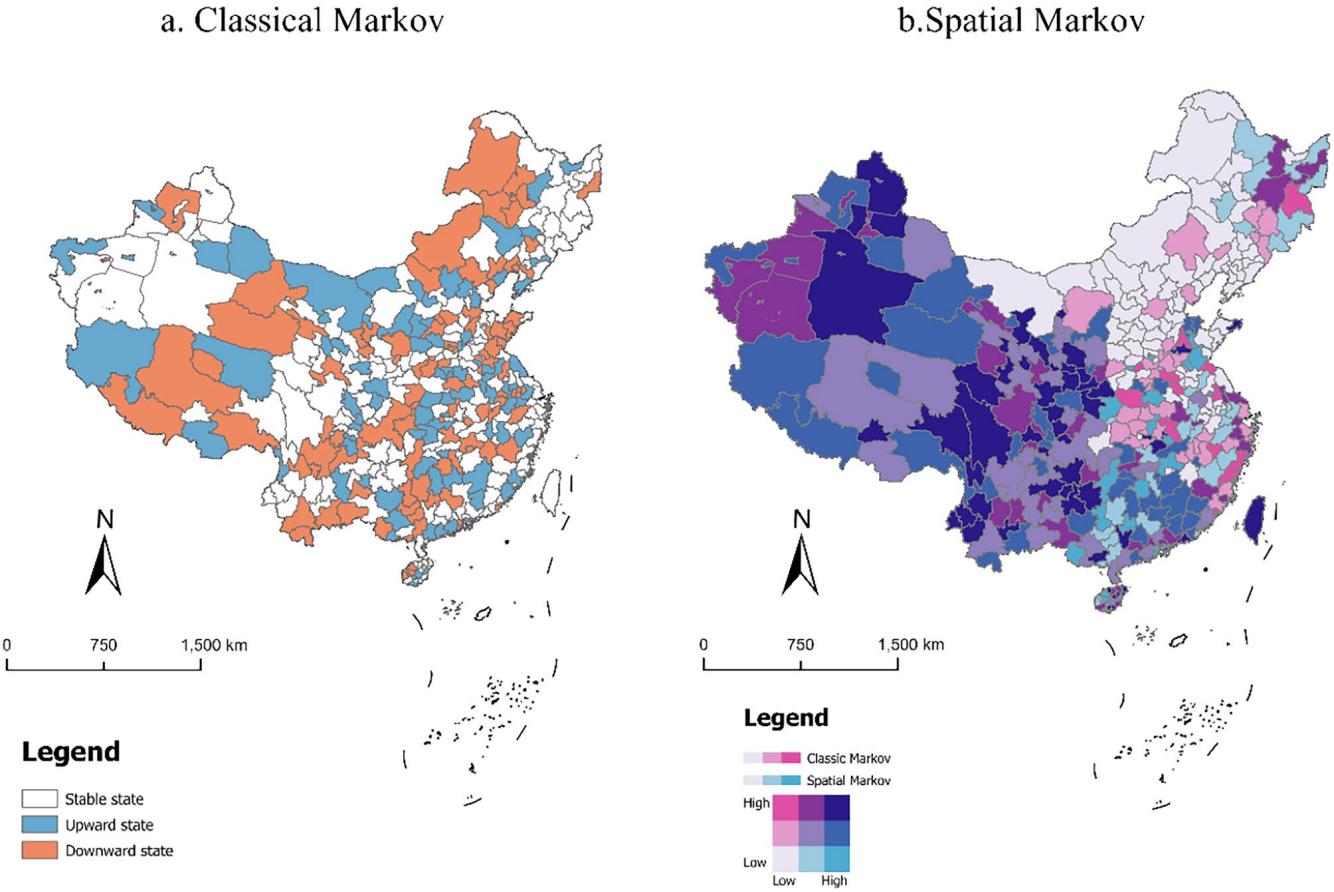
Spatial distribution pattern of TGIE type and neighborhood transfer of China from 2000 to 2020.

The results of the Markov transfer matrix showed that (1) the larger efficiency values at all stages were concentrated on the main diagonal and the efficiency value on the non-diagonal is less than the efficiency value on the diagonal, indicating that the transfer probability between different efficiency levels was relatively small and only an upward or downward transfer of a stage, suggesting it was difficult to have jumping transfers (e.g., jumping from a low-efficiency to a high-efficiency state). In other words, the TGIE change was a cyclical and gradual process, short periods of time had strong stability, and it was difficult to realize leapfrog development. (2) There were differences in the probability of transferring efficiency values at different stages. These results indicated there was a strong “club” phenomenon of TGIE convergence and within TGIE, suggesting significant endogenous evolutionary characteristics. (3) The high efficiency type had the strongest stability, with the probability of high efficiency remaining unchanged during the whole test period and having a prominent “club convergence” characteristic. Therefore, it was necessary to continuously improve the TGIE level of the middle efficiency region and promote it to join high-level clubs to avoid the “Matthew effect”. (4) From the spatial distribution map of TGIE type transfer (Fig. 5a), we found that there were 18 regions where TGIE maintained the smoothness of “club convergence”, with most regions in the east and a few regions in the west with this type of transfer. There were 12 regions with upward transfer and relatively centralized distribution, with the central and western region as the main regions. Downward adjustment was also possible and the probability of upward improvement, indicating it was relatively difficult to improve efficiency in a short time period. The number of upward shifting regions was larger than the number of downward adjustments, indicating that the TGIE level gradually increased in most regions and that the tourism industry was gradually transforming towards green development. With the rapid development of tourism in various regions, tourism exchanges between regions also became closer, suggesting the level of green innovation in tourism in neighboring regions will have a greater impact on local areas. Therefore, it is necessary to consider the impact of spatial factors, build a spatial Markov probability stochastic matrix (Table 3), and draw a spatial distribution map of TGIE-type transfers.

**Table 3:** The spatial Markov transfer matrix of TGIE in China from 2000 to 2020.

Typology	2000–2005			2005–2010			2010–2015			2015–2020		
	1	2	3	1	2	3	1	2	3	1	2	3
1												
1	0.750	0.351	0.767	0.644	0.581	0.343	0.17	0.864	0.306	0	0.363	0.300
2	0.102	0.665	0.17	0.464	0.23	0.224	0.348	0.106	0.726	0.063	0.055	0.766
3	0.548	0.197	0.056	0	0.401	0.625	0	0.851	0.349	0	0.376	0.303
2												
1	0.679	0	0.346	0.479	0.389	0	0.878	0.787	0	0.763	0.847	0.022
2	0	0.617	0	0.785	0.763	0.1	0.713	0.616	0.971	0.217	0	0.372
3	0.162	0.109	0.84	0	0.651	0	0.273	0.748	0.748	0.04	0.737	0.498
3												
1	0.97	0	0.034	0.287	0	0.878	0.922	0.943	0.355	0	0.092	0.520
2	0.728	0.902	0.450	0.679	0.249	0.308	0	0.662	0.208	0.540	0.169	0
3	0	0.732	0.064	0	0.128	0.378	0.763	0.939	0	0.195	0.863	0.597

From Fig. 5b, it can be observed that (1) each regional TGIE was in the upgrading stage and the surrounding areas are also shifted upward, with a good developmental trend. These areas where both the self and the neighboring areas are kept steady accounted for most of the areas and were concentrated in the eastern coastal regions. (2) Comparing the results presented in Tables 2 and 3, we found that there was an obvious spatial spillover effect in TGIE, with high-efficiency areas having a significant positive spillover effect on their neighboring areas and low-efficiency areas also having a negative spillover effect, forming a “club convergence” phenomenon in which high and low were clustered together and verified the kernel density estimation. Specifically, from 2000 to 2020, the probability of upward transfer was smaller than the probability of upward transfer of low efficiency in the traditional cases when low-efficiency types were neighbors with low efficiency. (3) There was asymmetry in the influence of neighborhoods on the situation at each stage. In 2000–2010 and 2010–2013, the average probability of inefficiency upward transfer under the influence of neighborhoods was greater than the average probability of high efficiency downward adjustment. In 2013–2020, the average probability of inefficiency upward transfer of is smaller than the average probability of high efficiency downward adjustment. The TGIE level in the previous two stages was in a period of fluctuating adjustment, and when the inefficiency of a neighbor was medium and high efficiency, the probability of the upward shift was greater than the risk of high efficiency downward adjustment via the neighbor influence. When TGIE was at a high level, it was more difficult to promote a shift upward and there was a greater risk of downward adjustment, which was consistent with the temporal changes in TGIE and suggested that a synergistic increase in regional TGIE was a key factor in the overall increase in the level of green innovation in tourism.

### Analysis of the driving factors of green innovation efficiency in China’s tourism industry

#### Selection of influencing factors

Analyzing the characteristics of the Spatio-temporal evolution of the green innovation efficiency of China's tourism industry showed that there were obvious regional differences in the TGIE. In light of the new situation and problems facing the sustainable development of the tourism industry, we analyzed the external environment of green innovation in the tourism industry and revealed the spatio-temporal evolution mechanism of green innovation in the Chinese tourism industry under the influence of multiple factors. Comprehensive existing research and available data on the social and cultural, economic development, and ecological environment aspects of the tourism industry were used to select six indicators as influencing factors^45^. These factors were the level of economic development (rgdp, per capita GDP), industrial structure (is, tertiary industry accounted for the proportion of GDP), the degree of technological innovation (tec, the number of domestic patent authorizations per 10,000 people), governmental regulation (er, ratio of environmental pollution control to fixed asset investment), degree of openness to the outside world (open, ratio of total regional import and export trade to GDP), and education level (edu, number of students enrolled in higher education institutions per million people). In addition, a Pearson test was conducted between TGIE and each influencing factor, which found that the correlation coefficients between the influencing factors and the explanatory variables were all greater than 0.5 and passed the confidence test of 0.01, indicating that these influencing factors have a certain rationality.

#### Stability tests

Before constructing the panel data regression model, all the variables were logarithmically processed. To avoid “pseudo-regression”, we further applied the unit root test methods of LLC, PP and ADF to test the smoothness of each variable (Table 4). We found that all the variables passed the significance test at the 10% level with at least two methods, which indicated that the panel data from the different regions had strong smoothness. The panel data regression model had three forms: random effect, fixed effect, and mixed effect. When the fixed effect regression model has a significant F statistic value, the model needs to be optimized for the mixed effect model. Second, the goodness of fit of the fixed and random effect models were compared using the results from the Hausman test (Table 5).

**Table 4:** The stationary test of panel data.

Shore	Test Methods	lnTGIE	lnrgdp	lnis	lntec	lner	lnopen	lnedu
Nationwide	LLC	− 87 (0.000)***	− 46.8 (0.016)**	− 75.3 (0.011)**	0.6 (0.017)**	13 (0.010)**	1.56 (0.010)**	32.5 (0.160)*
	ADF	− 20.8 (0.000)***	− 26.6 (0.012)**	− 49.2 (0.017)**	62.4 (0.000)***	8.7 (0.010)**	− 4.9 (0.018)**	29.7 (0.0133)**
	PP	− 23.6 (0.000)***	1.8 (0.019)**	− 63.8 (0.0136)**	− 3 (0.0184)**	55.9 (0.015)**	− 92 (0.000)***	7.2 (0.019)**
Eastern China	LLC	− 85.7 (0.312)	− 98.7 (0.011)**	1.2 (0.000)***	− 57 (0.012)**	92.1 (0.012)**	− 59 (0.013)**	− 74.5 (0.000)***
	ADF	51.8 (0.123)*	− 17.9 (0.000)***	16.6 (0.000)***	53.9 (0.000)***	− 30.5 (0.016)**	− 56 (0.000)***	22.8 (0.013)**
	PP	41.7 (0.000)***	− 43.2 (0.000)***	− 27.7 (0.107)*	− 4.7 (0.000)***	− 86.8 (0.000)***	− 46.6 (0.015)**	65 (0.013)**
Central China	LLC	− 94.8 (0.000)***	58.4 (0.000)***	42.8 (0.000)***	− 8.7 (0.017)**	− 89.9 (0.029)**	− 12.8 (0.017)**	− 6.9 (0.000)***
	ADF	− 51.1 (0.000)***	32.3 (0.000)***	− 85.9 (0.000)***	6.1 (0.000)***	48.7 (0.019)**	− 63.4 (0.000)***	− 63.9 (0.000)***
	PP	− 93.2 (0.000)***	− 17.4 (0.000)***	− 11.5 (0.000)***	80.6 (0.000)***	− 56.4 (0.000)***	− 48.7 (0.00)***	42.4 (0.015)**
Western China	LLC	− 38 (0.000)***	83.1 (0.000)***	− 94.4 (0.000)***	59.7 (0.000)***	− 8.3 (0.000)***	− 68 (0.000)***	43 (0.023)**
	ADF	− 7.9 (0.000)***	19	98.7 (0.000)***	− 19.7 (0.003)**	5 (0.000)***	5.7 (0.000)***	6.2 (0.000)***
	PP	− 76.2 (0.000)***	− 53	− 87.4 (0.0003)***	80.7 (0.000)***	− 51.5 (0.000)***	58.2 (0.000)***	25.8 (0.000)***

The P test values of regression parameters of each variable are in parentheses.

***, **, and *Indicate that the variables are significant at 1%, 5%, and 10% confidence levels, respectively.

**Table 5:** The regression results of the panel data model.

Variable	Nationwide		Eastern China		Central China		Western China	
	Fixed effect	Random effect	Fixed effect	Random effect	Fixed effect	Random effect	Fixed effect	Random effect
Constant term *c*	0.540** (0.09)	6.031 (0.357)	1.743** (0.293)	5.953 (0.770)	− 5.043 (− 0.508)	9.934 (− 0.508)	− 1.096 (− 0.061)	17.825 (0.951)
lnrgdp	− 0.104 (0.543)	− 0.191 (0.849)	0.017 (0.032)	0.536 (0.975)	− 0.599 (− 0.670)	0.894 (− 0.670)	− 0.086 (− 0.053)	1.605 (0.957)
lnis	0.303 (0.628)	0.483 (0.849)	0.473 (0.763)	0.620** (0.445)	− 0.306 (− 0.296)	1.035 (− 0.296)	0.007*** (0.004)	1.857 (0.997)
lntec	− 0.012** (0.016)	− 0.738 (0.629)	− 0.024 (− 1.531)	0.015 (0.126)	− 0.030** (− 1.163)	0.026** (− 1.163)	0.036** (0.775)	0.046 (0.438)
lner	0.003* (0.020)	0.148** (0.460)	0.005* (0.262)	0.020 (0.793)	0.032 (0.984)	0.033 (0.984)	0.002 (0.039)	0.059 (0.969)
lnopen	− 0.045* (0.040)	− 1.136 (0.883)	0.007 (0.177)	0.040* (0.859)	− 0.138* (− 2.096)	0.066* (− 2.096)	− 0.007 (− 0.059)	0.118 (0.953)
lnedu	0.053 (0.124)	0.429 (0.256)	0.075 (0.616)	0.122 (0.538)	0.360 (1.765)	0.204 (1.765)	− 0.180 (− 0.492)	0.366 (0.623)
Standard deviation	0.943	0.842	0.451	1.067	1.471	1.154	3.646	3.059
*R*^2^	0.357	0.961	0.483	0.084	0.558	0.760	0.152	0.537
Adjusted *R*^2^	0.011	0.023	0.205	0.315	0.320	0.087	− 0.305	0.925
F	1.032	7.130	1.738	0.185	2.346	14.427	0.333	0.118

The standard error of the estimated system is shown in parentheses.

***, **, and *Indicate that the variables are significant at 1%, 5%, and 10% confidence levels, respectively.

#### Mechanism analysis

The coefficients of GDP per capita for the entire country and the three regions were − 0.104, 0.017, − 0.599, and − 0.086, respectively. The level of economic development plays a positive role in promoting the eastern region, and a negative role in the central and western region. This shows that the higher the economic development, the more conducive to the development of TGIE. It can be seen that economic development also has a threshold effect on TGIE, and only when the regional economic development level crosses this threshold can it have a positive effect on TGIE.

In addition, the coefficient of the eastern region had a high degree of influence, while the central and western region played a weaker role owing to backward economic development. Indeed, only when there is enough regional economy development is there significant support of the industry and optimization of the environment that guarantees the green development of tourism. The coefficients of the proportion of tertiary industry were 0.303, 0.473, − 0.306, and 0.007, respectively; and western region passed the 5% level significance test, indicating a significant positive relationship between the optimization of industrial structure and TGIE. The type of industrial structure and the degree of development directly affected economic efficiency and resource utilization efficiency such that a high proportion of the tertiary industry characterizes the degree of regional attention to the development of the tourism industry, while a high level of tourism specialization indicates tourism economic efficiency and resource utilization efficiency will gradually improve and reduce the coercive impact on the ecological environment. The eastern region had the highest impact coefficient (0.473), consistent with the reform and opening up, energy construction, raw material industry, special agricultural product processing, and tourism becoming characteristic industries in the western region. The higher the proportion of tertiary industry, the more economic development in the western region changed from relying on the secondary industry with relatively high energy consumption and pollution, to the tertiary industry with relatively low pollution, further supporting the rapid development of tourism in recent years. In particular, the development of eco-tourism and other green industries have turned regional resource and regional advantages into economic advantages, reflected in the optimization of industrial structure having a more significant impact on TGIE relative to the middle and eastern region.

The coefficients of technological innovation were − 0.012, − 0.024, − 0.030 and 0.036, respectively; except for the eastern region, and all of them were significant at the 5% level, indicating a significant positive relationship between technological innovation and TGIE. Technological innovation plays an important role in reducing the waste of resources, reducing environmental pollution, and improving the efficiency of the green economy, as well as providing technical and environmental support for the green development of tourism; however, the role of technological innovation in TGIE varied in the different test regions. The decreasing role coefficient of the eastern, central, and western region was because the eastern region had a good economic foundation, a high level of modernization, and technological innovation concentrated in the tertiary industry, which contributed to the innovation of green technology, continuous improvement of the regional ecological environment, and had a greater impact on TGIE. To improve the urbanization level, the central and western region were focused on industrial production technology innovation, with less green technology innovation output and insufficient attention to environmental protection and governance, which had a relatively small impact on TGIE. The coefficients of government regulation in each region were 0.003, 0.005, 0.032, and 0.002, respectively, with only insignificant value observed in the whole country and eastern region. This indicated that the improvement in TGIE depended on the government's intervention in the tourism economy and tourism environment. The intensity of such an intervention can effectively promote the synergistic development of the tourism economy, society, and ecological environment within a reasonable range. However, the coefficient in the eastern region was 0.005, which indicated that in a region with a high degree of marketization, the market itself achieved efficient allocation of resources through competition, price, supply, and demand mechanisms, and that excessive macro-regulation was not conducive to the formation of an environmental economic system, such as the green capital market, green credit, or ecological compensation. Therefore, the government will need to combine the market with appropriate regulations and controls to contribute to the tourism industry's green innovation.

The coefficients of openness to the outside world for the different regions were − 0.045, 0.007, − 0.138, and − 0.007, respectively; Only the whole country and the central region passed the significance test. With the rapid development of the domestic tourism market, inbound tourists accounted for less than 1% of the market share for cities such as Beijing, Shanghai, and Guangdong, with a high degree of openness to the outside world. Foreign investment in the tourism industry is valued for China's rich tourism resources, cheap labor, and a broad tourism market, mostly for the purpose of economic benefits. The government may relax environmental regulations to attract foreign investment and put pressure on the ecological environment, reducing the significant effect on the relationship between opening up and TGIE. The coefficients of education level were 0.053, 0.075, 0.360, and − 0.180, respectively, for the tested regions, indicating that the improvement of education level was conducive to enhancing the level of TGIE and can reflect the academic quality of the general public in society, the awareness of stronger environmental protection, higher professional skills and innovation of the personnel engaged in the tourism industry, and stronger tourism innovation technology. The higher the professional skills and innovation ability of personnel engaged in the tourism industry, the stronger the tourism innovation technology and the better the tourism economic efficiency, resource utilization efficiency, energy saving, and emission reduction effects. The western region had a relatively low education level, and with the rapid development of the tourism industry in recent years, there was a relative lack of high-quality tourism practitioners. Therefore, the improvement of education level had a more significant impact on TGIE than in the eastern and central parts of the country. Overall, except for the role of opening up to the outside world, most of the influencing factors effectively improved regional TGIE levels to some extent; however, there were obvious regional differences among these factors.

## Conclusion and discussion

### Conclusion

To define the implications of green innovation efficiency on the tourism industry, we constructed an input–output index system using an SBM-undesirable model to comprehensively evaluate the green innovation efficiency of China's urban tourism industry. Using exploratory spatial analysis and spatial econometric models, we revealed Spatio-temporal evolution rules and influencing factors: (1) In general, the efficiency of green innovation in China's urban tourism industry was low and there is room for improvement. From 2000 to 2020, the green innovation efficiency of China's urban tourism industry was at a medium level, showing a “W” shape of “decline–rise–decline–rise”. Green innovation efficiency showed a rising fluctuation trend in the entire country, transition in the east, collapse in the middle, and stagnation in the northeast. (2) In terms of dynamic evolution, TGIE always exhibited polarization, but regional coordination was gradually enhanced by strong stability and it was difficult to achieve leap-forward development. The cities with spatial upward transfer were concentrated mainly in the central and western region, while there are few cities with downward adjustment. There were obvious spatial spillover effects, which were asymmetrical. (3) In terms of driving factors, the overall economic level, industrial structure, government regulation, and education levels had a significant positive relationship with TGIE, while the degree of opening up to the outside world had no significant effect, although the degree of influence, mechanism, and conditions of each factor were strongly regional.

### Discussion

To further improve the efficiency of green innovation in the tourism industry and promote coordinated development among cities, the following policy recommendations are put forward. First, the overall concept and systematic thinking should be established to promote the coordinated development of the green innovation efficiency of the tourism industry in different cities. Through the establishment of a support mechanism, efficient cities should be encouraged to provide support to inefficient cities and the flow of knowledge, talent, and other resources among different cities should be accelerated. Indeed, the green innovation efficiency network of the tourism industry has produced linkage and coordination effects during the transmission process^46^. Second, from the perspective of each city node and its formed plates, attention should be paid to breaking the Matthew effect on the green innovation efficiency of the tourism industry so that all cities can achieve a balance between reception and spillover relationships. Cities in the central and western region should minimize their spillover effect and balance the development of green innovation efficiency of the tourism industry in all cities as a primary driver of innovation for the eastern region. Within the reasonable transfer and transmission costs of green innovation efficiency, eastern cities should play a leading role in the central and western cities, promote the balanced development of tourism in various regions, optimize the transmission mechanism of green innovation efficiency of the tourism industry among different sectors, promote regional linkage and collaborative improvement among various sectors, and promote green innovation and sustainable development of China's tourism industry. In addition, the improvement and development of tourism transportation infrastructure should be accelerated, the distance between cities should be shortened, the status of cities in the central and western region in the green innovation efficiency space network should be improved, and regionally balanced and coordinated development of green innovation in China's tourism industry should be promoted.

## Data Availability

The datasets generated during and/or analysed during the current study are available from the corresponding author upon reasonable request.
